# Filling the Silent Void: Genetic Therapies for Hearing Impairment

**DOI:** 10.1155/2012/748698

**Published:** 2012-12-04

**Authors:** Joel Sng, Thomas Lufkin

**Affiliations:** Stem Cell and Developmental Biology, Genome Institute of Singapore, 60 Biopolis Street, Singapore 138672

## Abstract

The inner ear cytoarchitecture forms one of the most intricate and delicate organs in the human body and is vulnerable to the effects of genetic disorders, aging, and environmental damage. Owing to the inability of the mammalian cochlea to regenerate sensory hair cells, the loss of hair cells is a leading cause of deafness in humans. Millions of individuals worldwide are affected by the emotionally and financially devastating effects of hearing impairment (HI). This paper provides a brief introduction into the key role of genes regulating inner ear development and function. Potential future therapies that leverage on an improved understanding of these molecular pathways are also described in detail.

## 1. Introduction

The human ear is a highly complex instrument that is comprised of three main sections: the outer ear, the middle ear, and the inner ear. While many surgical remedies exist for the treatment of hearing loss stemming from dysfunction of the outer and middle ear, few effective remedies have been developed for the treatment of hearing impairment resulting from inner ear disorders. The inner ear is comprised of two main components, the auditory system which receives the amplified mechanical vibrations transmitted from the middle ear and the vestibular system which is responsible for maintaining balance. The auditory system of the inner ear consists of the cochlea which contains three fluid filled spaces, the scala vestibule, scala media, and scala tympani [1]. Mechanical vibrations from the middle ear are propagated through these spaces and are detected by the organ of Corti which is located on the basilar membrane of the scala media. The organ of Corti is a complex structure containing hair cells and supporting pillar and Deiters cells. Hair cells located in the organ of Corti generate action potentials in response to perturbations which are transmitted to the auditory cortex via the cochlear nerve [2].

Hearing loss is classified according to the region of the ear affected. Conductive hearing loss is usually diagnosed as defects in the outer and middle ear that prevent sound from being transmitted to the cochlea while sensorineural hearing loss is usually diagnosed as dysfunction of the inner ear, cochlear nerve, or auditory cortex. Many studies have demonstrated that the impact of inherited genetic mutations on hearing impairment (HI) is especially significant. Various mutations in a single gene can cause either syndromic or nonsyndromic hereditary hearing loss (HHL) and result in HI at different stages in life and over seventy chromosomal genes. Two mitochondrial genes, which harbour seven different mutations, have been linked to nonsyndromic HHL alone [3]. HHL also is the main cause of early-onset HI with more than 60% of such affected individuals suffering from HHL [4], with single-gene mutations probably accounting for at least half the cases of childhood deafness [5, 6]. In addition, about ten percent of the adult population is affected by HI [7] and age related HI is one of the most prevalent chronic conditions with 25–40% of the population aged 65 or older affected [8]. As the global population ages, research on developing preventive medicine and therapies for HI is increasingly essential. A majority of these patients suffer from hearing loss owing to damage to hair cells in the cochlea [9] and as these cells do not regenerate spontaneously in mammals, the loss of hearing is often permanent [10, 11]. Hence understanding the link between genetics, HI, hair cells, and the highly differentiated supporting cells surrounding them, remains a prerequisite to developing effective regenerative therapies.

## 2. The Role of Genetics

A concerted effort of many genes is required for the development, maintenance, and proper functioning of the inner ear and a comprehensive mastery of these molecular pathways is essential for understanding how inner ear progenitor cells progress through states of developmental competence, coordinated cell cycle exit, and differentiation to form and maintain the cochlea's complex cytoarchitecture. To this end, animals have served as attractive models of human HI owing to the difficulty of observing key developmental pathways and the progression of dysfunction in the human cochlea [4, 12, 13] (Table 1). The homeobox gene family is one of the major groups of genes that play an important role in inner ear development and has been extensively studied in various animal models. Characterized by a 180 bp homeodomain, these genes encode for essential transcription factors that can recognize and bind to specific DNA motifs and act as key regulators of morphogenesis [14]. Many members of the homeobox gene family have been implicated in vertebrate inner ear formation including the *Pax* paired-homeobox gene family, *Otx* homeobox gene family, *Gastrulation brain homeobox (Gbx)* gene family, *Msx* homeobox gene family, *Dlx* homeobox gene family, and *Hmx* homeobox gene family (for review see [15]).

Other genes play equally important roles in ensuring correct cellular patterning in the inner ear. The *Notch* signaling pathway and its ligands DLL1 and JAG2 form a highly conserved cell signaling system essential for influencing the fate of progenitor cells during inner ear formation and lateral-inhibition mediated differentiation of hair cells [48]. High levels of *Notch* signaling promote *Sox2* expression [52] which may encourage the initial proliferation of inner ear stem cells to form a prosensory zone of nonproliferating cells expressing inhibitory p27^Kip1^ along the length of the cochlea. This expression of *Notch1* also inhibits premature hair cell differentiation. A combination of the *Notch-Hes1* pathway and *Fibroblast growth factor* (*Fgf*) signaling is also essential for activating various transcription factors required for the further specification in the prosensory domain of the inner ear potentially by the transcriptional downregulation of p27^Kip1^ [50, 53, 54]. Later reduction of *Notch* signaling then increases *Atoh1* (also known as *Math1*) expression which induces the formation of hair cells [55]. Initially differentiated inner hair cells can then direct the secondary differentiation and placement of neighboring supporting cells such as the pillar cells. Inner hair cells express *Fgf8* and produce Fgf8 which is a high affinity ligand of Fgf receptor 3 found on neighboring progenitor cells [56, 57]. Binding of Fgf8 to Fgfr3 induces the differentiation of these neighboring cells into pillar cells forming the distinct cellular patterns in the cochlea [40, 58]. The production of Fgf8 in hair cells also maintains the expression of *Hey2* in surrounding pillar cells which prevents them from transdifferentiating into hair cells and creates a clean distribution between pillar cells and Deiters' cells by inhibitory interactions between *Hey2* and *Hes5*, preserving the complex patterned structure of the cochlear [47]. Hence *Notch* signaling plays an essential role in regulating the expression of transcription factors and aids in the differentiation and maintenance of inner ear stem cells to form the key cell types in the cochlea.

The *retinoblastoma* (Rb) family of cell cycle regulators such as *Rb1* (pRb), *Rbl1* (p107), and *Rbl2* (p130) also plays an essential role in regulating the proliferation of supporting cells and hair cells [23, 27, 59]. In particular, *Rb1* (and its encoded protein pRb) plays an essential role in hair cell quiescence and pRb inactivation results in cell cycle reentry and the abnormal proliferation of hair cells [24–26]. pRb functions by interacting with and inhibiting the activity of E2F transcription factors such as E2F1 [38, 39]. However while the loss of pRb is most significant during the early phases of inner ear development and leads to increased proliferation of hair and supporting cells, Rb1 deletion alone in the adult mouse is insufficient to reinitiate proliferation in the inner ear suggesting that other regulators are able to compensate for the loss of Rb1 [23]. 

The above description of the *Notch1* signaling pathway, Rb cell cycle regulators, and homeobox genes only provides an abridged version of the complex web of gene regulatory networks necessary for transforming a mass of undifferentiated stem cells into the complex cytoarchitecture of the inner ear. There is substantial evidence that the *Myosin* gene family plays a critical role in the function of inner ear hair cells. Myosins are a superfamily of ATP-dependent motor proteins which can act as force sensors to detect auditory stimuli [60] and are divided into at least twenty-four classes [61]. Various myosins play significant roles in hearing. Myosin genes implicated in human hearing loss include *MYO1A*, *MYO6*, *MYO7A*, and *MYO15*. The myosin-I isoform (myo1c) has been shown to sensitize transduction channels and is an essential component of the hair cell's adaptation-motor complex [62, 63] in mice. Mutations in *MYO1A,* a cochlear expressed gene located in the DFNA48 locus, have also been identified as a contributor to autosomal dominant hearing loss in humans [28]. Other genes of interest include *Forkhead* genes like *Foxg1* which can cooperate with *Fgf10* and interact with the *Notch/Hes* signaling system to regulate the size of sensory epithelia [43], and basic helix-loop-helix genes like *Ngn1* that promote neurogenesis and maintain progenitor cell populations [49] (Table 1).

## 3. Developing Therapies for HI

Although it is relatively easy to diagnose HI, it is much harder to determine its underlying causes owing to their large heterogeneity ranging from complex genetic disorders, environmental effects, drug-side effects, infection, and other unknown causes. In addition the only available treatment options are limited to hearing aids and cochlear implants which are not equally effective in all patients owing to the complex pathogenesis of HI and different degrees of tissue damage. There are also no treatments available to arrest or reverse the progression of HI. In the light of these limitations, there has been intense interest in developing new approaches for treating HI which include developing gene therapies, stem cell therapies, and drugs to induce the regeneration of the sensory epithelia in the inner ear. To overcome the difficulties of studying degenerative changes in the human cochlea, several animal models that replicate the symptoms of HI have been developed. These include the development of in utero gene transfer mouse models [64], for evaluating the effectiveness of potential gene therapies in reversing human HI [4, 65].

Accurate diagnosis of the underlying cause of HI on a molecular level will be essential for the design of personalized gene therapies since many different gene mutations can cause similar HI phenotypes. The search for new targets for human HI gene therapy is hindered by the lack of tools to study inner ear function *in vivo* as well as cell lines which accurately model cochlea function. Hence mice and guinea pigs remain popular model systems of human HI owing to their evolutionary closeness and the availability of many hearing-impaired mutant lines. Gene therapy which focuses on the regeneration of damaged hair cells by restoring the expression of *Atoh1* (or *Math1*) is commonly seen as one of the more promising candidate cures for HI. *Atoh1* encodes for a helix-loop-helix transcription factor which is required for the differentiation of hair cells in the vertebrate inner ear and targeted disruption of *Atoh1* prevents the development of auditory and vestibular hair cells [66]. Targeted expression of *Atoh1* in the organ of Corti is also essential for normal hair cell function and maintenance. Delayed *Atoh1* deletion in conditional knockout mice causes the loss of inner hair cells and a significant amount of outer hair cells three weeks after delivery [36]. Reexpression of *Atoh1* by *in vivo* adenovirus mediated gene transfer has been shown to induce the regeneration of hair cells, encourage neuronal growth towards these newly differentiated cells, and reverse HI in adult Guinea pigs [37, 67]. These cells have also been characterized by patch clamping and been shown to have functional mechanotransduction [64]. Hence gene therapy involving *Atoh1* may potentially be a viable method for restoring the damaged auditory neuroepithelium. However, reexpression of *Atoh1* alone may not be sufficient to improve hearing thresholds if endogenous progenitor cells are absent [68]. The effectiveness of direct transdifferentiation of supporting cells to hair cells may also be limiting because of the loss of supporting cells that have equally important roles, insufficient numbers of transdifferentiated hair cells generated, and the nonideal positioning of newly transdifferentiated hair cells. Hence the complex multifactor nature of regeneration requires combinatorial gene therapies that simultaneously guide proliferation, transdifferentiation, and positioning of progenitor cells to restore the cytoarchitecture structure and function of the organ of Corti. For example, the overexpression of the cell cycle enhancement gene *SKP2* can induce proliferation of nonsensory cells which can then transdifferentiate in the presence of *Atoh1* [69]. This combined overexpression of both *Skp2* and *Atoh1* induces formation of a greater number of hair cells compared with *Atoh1* overexpression alone and indicates that multiple gene therapies may provide more effective solutions for reversing the effects of HI.

For targeted gene therapy to be successful, the identification of progenitor cells for hair cells and the elucidation of molecular pathways that regulate their maintenance, proliferation, and specialization are essential. These cells are necessary as they have the potential to differentiate or transdifferentiate into hair cells. Identification of gene targets that maintain and regulate these cells will be essential for reversing the symptoms of HI. Potential progenitors of hair cells may be found in the dorsal epithelium of the cochlear canal [70] and could be induced to differentiate into functional hair cells. Pluripotent stem cells have also been discovered in the adult utricular sensory epithelium and are able to form cells of all three germ layers, including hair cells [71]. Supporting cells could also be a potential target in future gene therapies as they serve as a natural source of new hair cells in nonmammalian vertebrates [72–74] and have also demonstrated limited capacity for transdifferentiation in some mammalian studies [75, 76].

Gene therapies could be combined with cochlear implants to develop novel cures for HI. While spiral ganglion density in most patients who receive cochlear implants may initially be sufficient to produce satisfactory results, the long term effectiveness of cochlear implants which operate by exciting spiral ganglion neurons (SGN) for patients with profound sensorineural hearing loss is limited because of the potential degradation of SGNs [77, 78]. The loss of hair cells and supporting cells which produce neurotrophins like NT-3 [79] and maintain the SGN via the neuregulin (NRG)-erbB receptor signaling pathway [80] contributes to the degradation of the SGN and neural death, however the continuous inoculation of neurotrophins to the cochlea can halt SGN degradation in deaf guinea pigs [81, 82]. Fibroblast growth factors (FGFs) are also essential for SGN maintenance [83] and combined treatment with FGF and neurotrophins may prevent secondary deterioration of SGNs [84]. Hence gene therapy stimulating the *in vivo* production of neurotrophins and fibroblast growth factors combined with electrical stimulation from cochlear implants could encourage extended survival of SGNs by inducing long-term *in vivo* production of essential growth factors and improve the long-term therapeutic benefits of cochlear implants [85, 86]. Therapies which maintain neurotrophin producing supporting cells populations would also have similar effect [87].

Therapies involving the transplantation of exogenous stem cells and other multipotent cells also provide a possible solution for the reconstitution of normal cochlea function if endogenous progenitor cells are absent. Stem cells have the capacity for self-renewal and are able to form specialized cell types including hair cells, spiral ganglion neurons, and their progenitors [88] for restoring normal cochlea function. Many studies have demonstrated the innate ability of transplanted cells to survive and differentiate. For example, the introduction of bone marrow stromal cells into the cochlea of chinchillas has resulted in increased expression of neuronal and glial cell markers in grafted cells suggesting their potential as transplants for restoring cochlea function [89]. Previous studies have also proven that fetal mouse and guinea pig spinal ganglions can survive grafting into the cochlea of their adult counterparts and that the survival of these implants is increased by treatment with neurotrophic factors like ciliary neurotrophic factor and brain-derived neurotrophic factor [90, 91]. The combined treatment of implanted cells with neurotrophic growth factor and chronic electrical stimulation also stimulates increased neurite outgrowth to the spiral ganglion region [92]. In addition, xenografts of the spinal ganglion neurons, embryonic stem cells, and adult neural stem cells (in rat and guinea pig) can survive, migrate, and differentiate successfully in the relatively immunoprivileged nature of the peripheral and central nervous system [93]. More recently, the implantation of hESC-derived neurons and hair cells into auditory neuropathic gerbils led to the successful recovery of auditory neuron functionality and the restoration of auditory evoked response thresholds [94]. These studies demonstrate the potential of transplants to restore normal cochlea function and form neuronal connections between the cochlea and the central nervous system. In addition the possibility of developing combinational therapies involving stem cell transplants, cochlear implants, gene therapy, and drugs could also lead to an effective therapeutic solution for a wider range of hearing impaired patients.

The development of novel techniques for efficient delivery of emerging therapies continues to be an essential component of a successful therapy. Advances in this area have led to the refinement of procedures for stem cell transplant, gene therapy, and controlled local drug delivery. Potential stem and progenitor cell transplants could be performed surgically via the basal turn of the cochlea or through implantable delivery systems (for review see [95]). Successful stem cell transplantation will also involve the use of either a microinjector or an osmotic pump coupled with a catheter system enabling multiple implantation of small volumes of cells at specific locations within the inner ear [96, 97]. While the various techniques developed allow the implantation of therapeutic cells into various sites of the inner ear (scala media, scala tympani, perilymphatic space, and modiolus) key limitations preventing the utilization of existing techniques in successful therapies include the fact that transplanted cells often fail to integrate appropriately with epithelium architecture and invasive surgical procedures may result in the loss of endolymph from the scala media resulting in the disruption of cochlear function [95, 98].

The effectiveness of various viral and nonviral vectors has been investigated for inner ear gene therapy. For example, bovine adeno-associated virus vectors (BAV) inoculated to the scala tympani resulted in successful transgene expression at the membranous labyrinth [99]. Supporting *in vitro* research also indicates that BAV can be successfully utilized for restoring gap junction coupling and connexin protein expression [100]. The microinjection of adenovirus based vectors near the fenestra cochleare also resulted in sustained protein expression for approximately four weeks [101]. Recent studies have also demonstrated the potential of nonviral vectors in gene therapy. Hyperbranched polylysine nanoparticles administered to the round window of rats were able to integrate efficiently into spiral ganglion cells and organ of Corti [102]. Nerve Growth Factor-derived peptide functionalized nanoparticles may also be a viable tool for targeted drug delivery into the inner ear [103]. Similarly biodegreadable CGP-hydrogels, liposome, and polymersome nanoparticles can be synthesized and injected onto the round window niche for controlled delivery of drugs to the inner ear [104, 105]. The increasing availability of specialized instruments such as microendoscopes, cochlear implant associated delivery systems, and reciprocating drug delivery systems presents an expanding variety of options for drug delivery and increases the potential that similar methods could also be utilized for gene therapy and progenitor cell transplants [106–108].

## 4. Conclusion

Animal models of human HI remain essential for investigating potential future therapies that leverage on an improved understanding of the molecular pathways that regulate proliferation, differentiation, and structure in the inner ear. To cure HI due to dysfunction of the inner ear, therapies that induce functional restoration of the highly complex cochlea cytoarchitecture must be developed. Future combinational therapies involving various permutations of progenitor cell transplantation, cochlear implants, targeted gene therapy, and drugs may provide interesting therapeutic results and lead the development of effective therapies for a wider range of HI patients.

## Figures and Tables

**Table 1:** 

Genes that regulate the development of the ear		
Gene	Description	References
Homeobox gene superfamily		

*Pax *	*Pax2* required for organ of Corti formation, expression of *Pax5* can compensate for loss of *Pax2 *expression.	[16]
*Otx *	*Otx1* required for saccule and utricle segregation and formation of the horizontal canal. *Otx2* required for segregation of inner ear and trigeminal progenitors.	[17, 18]
*Gbx *	*Gbx2 *required in posterior otic placode formation.	[18]
*Msx *	*Msx1* expressed in the preotic placodal region, possibly a regulator of neural development. Plays role in regulating epithelial-mesenchymal interaction.	[15, 19, 20]
*Dlx *	*Dlx5 *and *Dlx6* required for formation of semicircular ducts, saccule, and utricle. Inhibits *Pax2* and activates *Gbx2* expression.	[21]
*Hmx *	*Hmx2 *and *Hmx3* coexpressed in the dorsolateral otic epithelium, controls cell proliferation, and regulates morphology of inner ear.	[22]

Retinoblastoma (Rb) family		

*Rb1 *	Cell cycle regulator (G to S phase transition), required for hair cell quiescence. Deletion of *Rb1* induces proliferation and differentiation of hair cells. However deletion of *Rb1 *in adult mice is insufficient for inducing hair cell proliferation.	[23–26]
*Rbl1 *	Cell cycle regulator (G to S phase transition).	[24]
*Rbl2 *	Cell cycle regulator (G to S phase transition). Deletion results in additional rows of hair and supporting cells.	[27]

Myosin superfamily		

*MYO1A *	Located within DFNA48 locus, expressed within cochlear, mutation results in sensorineural hearing impairment. Myo1b located at apical surface of supporting cells, Myo1c concentrated at hair cell stereocilia, Myo1e located at hair cells of auditory epithelia.	[28, 29]
*MYO6 *	Required for the structural maintenance of hair cell stereocilia, mutation leads to autosomal dominant hearing loss.	[30, 31]
*MYO7A *	Required for inner ear endocytosis, mutations can result in Usher syndrome or nonsyndromic deafness.	[32, 33]
*MYO15 *	Required for development and elongation of hair cell stereocilia, mutation associated with hearing impairment.	[34, 35]

Other genes		

*Atoh1 (Math1) *	Helix-loop-helix transcription factor required for the development, differentiation, and regeneration of functional hair cells.	[36, 37]
*E2F1 *	Transcription factor involved in cell cycle regulation. Mediates pRb (*Rb1*) function.	[23, 38, 39]
*Fgf *	Expression of *Fgf3* and *Fgf10* required for *Dlx5* and *Dlx6 *expression. *Fgf10 *required for posterior canal and hair cell cilia formation. *Fgf8 *binds to *Fgfr3* and is involved in pillar cell formation and cellular patterning in cochlea.	[40–42]
*Foxg1 *	Required for inner ear sensory cristae formation, regulates sensory fate and embryonic neurosensory development. Mutation results in a shortened cochlea and loss of crista neurons.	[43–45]
*Hes1 *	Controls patterning of inner hair cells in organ of Corti, functions with *Hey2*.	[46]
*Hes5 *	Controls patterning of outer hair cells in organ of Corti, functions with *Hey2*.	[46]
*Hey2 *	Activated by *Fgf*, prevents pillar cells from differentiating into hair cells. Controls patterning in organ of Corti, functions with *Hes1* and *Hes5*.	[46, 47]
*Notch *	Regulates hair cell fate and patterning in cochlea, regulated by *Notch* ligands DLL1 and JAG2.	[48]
*Ngn1 *	Regulates transition from neurogenesis to sensory cell development. Process cross-regulated by both *Ngn1* and *Atoh1*.	[49]
*p*27^*Kip*1^	Regulated by *Notch-Hes1 *signalling pathway. Cyclin-dependent kinase cell cycle inhibitor. Loss of *p*27^*Kip*1^ initiates cellular proliferation in organ of Corti.	[50, 51]
*Sox2 *	Promoted by Notch1 signalling, regulates hair cell differentiation and proliferation. Mutation results in sensorineural hearing impairment.	[52]
